# ComQXPA Quorum Sensing Systems May Not Be Unique to *Bacillus subtilis*: A Census in Prokaryotic Genomes

**DOI:** 10.1371/journal.pone.0096122

**Published:** 2014-05-02

**Authors:** Iztok Dogsa, Kumari Sonal Choudhary, Ziva Marsetic, Sanjarbek Hudaiberdiev, Roberto Vera, Sándor Pongor, Ines Mandic-Mulec

**Affiliations:** 1 Department of Food Science and Technology, Biotechnical Faculty, University of Ljubljana, Ljubljana, Slovenia; 2 Group of Protein Structure and Bioinformatics, International Centre for Genetic Engineering and Biotechnology, Trieste, Italy; 3 Faculty of Information Technology and Bionics, Pázmány Péter Catholic University, Budapest, Hungary; Rockefeller University, United States of America

## Abstract

The *comQXPA* locus of *Bacillus subtilis* encodes a quorum sensing (QS) system typical of Gram positive bacteria. It encodes four proteins, the ComQ isoprenyl transferase, the ComX pre-peptide signal, the ComP histidine kinase, and the ComA response regulator. These are encoded by four adjacent genes all situated on the same chromosome strand. Here we present results of a comprehensive census of *comQXPA*-like gene arrangements in 2620 complete and 6970 draft prokaryotic genomes (sequenced by the end of 2013). After manually checking the data for false-positive and false-negative hits, we found 39 novel *com*-like predictions. The census data show that in addition to *B. subtilis* and close relatives, 20 *comQXPA*-like loci are predicted to occur outside the *B. subtilis* clade. These include some species of Clostridiales order, but none outside the phylum Firmicutes. Characteristic gene-overlap patterns were observed in *comQXPA* loci, which were different for the *B. subtilis*-like and non-*B. subtilis*-like clades. Pronounced sequence variability associated with the ComX peptide in *B. subtilis* clade is evident also in the non-*B. subtilis* clade suggesting grossly similar evolutionary constraints in the underlying quorum sensing systems.

## Introduction

*B. subtilis* is one of the most studied prokaryotes and a frequently used model organism for Gram positive bacteria. It is capable of secreting a wide variety of molecules, including ribosomally and non-ribosomally produced peptides, polyketides, etc (for a recent review see [1], [2]). Some of these play the role of quorum sensing (QS) molecules that coordinate social response of various bacterial populations in a cell density dependent manner [3]. Here we focus on the major *B. subtilis* quorum sensing *comQXPA* locus that operates through the ribosomally synthesized signaling peptide ComX. This peptide, which is modified by the ComQ isoprenyl transferase, specifically binds to the membrane receptor ComP, which then through phosphorylation of the response regulator ComA, elicits the QS response genes [4]–[10]. Note that we use the term “quorum sensing” with no implication of the relative importance of diffusion, flux, evolutionary and ecological roles (for recent reviews see [2], [11]–[13]). We also use the term “signal” without reference to evolutionary adaptation, but simply to denote a chemical compound that alters the functioning of a bacterial cell.

ComX was first identified 20 years ago as a pheromone molecule present in the supernatant of high density cultures of *B. subtilis* [9], [14]. Upon reaching a critical concentration it activates a large number of cellular responses including competence development, surfactin production, biofilm formation and extracellular DNA release [13], [15]–[17]. Current research shows that ComX plays a pivotal role in activating cellular differentiation of *B. subtilis* in that ComX producing cells activate quorum sensing response only in a subpopulation of cells [4], [18]–[20]. The QS response itself is regulated by a negative feedback loop that is based on production of ComX and sensed privately by the producing cell [19]. Another direction of studying subpopulations is the comparison of *B. subtilis* undomesticated strains isolated from the environment. These show high rates of polymorphisms of the ComQXPA quorum sensing system. The polymorphisms are reflected in functional diversification of *B. subtilis* strains into four different pherotype groups. These groups consist of strains among which effective communication is possible, but where communication across pherotypes is impaired [7], [18], [21], [22]. Most recently, it was also found that pherotype diversification is present even within 2.5 mm^3^ soil samples [23] and correlates, although imperfectly, with ecotype diversification [24].

The functional importance of the ComQXPA quorum sensing system and its interspecific polymorphisms make it a very interesting model system to study. The functioning of the ComQXPA quorum sensing system in *B. subtilis* relies on four proteins, ComQ, ComX, ComP, and ComA that are encoded by four adjacent reading frames situated on the same strand of the *B. subtilis* chromosome [7]. There is one common promoter in front of *comQ* and two putative promoters - one in front of *comX* and a second in front of *comA* [4]. We briefly overview the functional properties of the four proteins in the following paragraphs.

### ComQ

A notable property of the ComQXPA QS system is that it requires an enzyme for postranslational modification of the ComX prepeptide. This is achieved by ComQ, which in *B. subtilis* is 286 to 309 amino acids in length (average length 299). It functions as an isoprenyl transferase and has the unique ability to attach the isoprenyl units to the tryptophan residue of ComX signal peptide. Isoprenylation occurs via an unusual ring-like structure formed upon addition of a farnesyl or geranyl group [25]. The sequence of the peptide pheromone and type of isoprenylation can vary from strain to strain. For instance, *B. subtilis* RO-E-2 uses an active pheromone of only five residues in length containing a geranylated tryptophan [25], [26].

All *B. subtilis* ComQ protein sequences bear similarities to isoprenyl phosphate synthetases in general [4]. In domain databases, ComQ is reported to be a single-domain protein (e.g., PFAM: PF00348.12), with some databases (UNIPROT) indicating a putative membrane-binding segment of the protein. Consistent with such observations, some sequence-based predictions indicate that ComQ may have a membrane bound segment [8]. However, these predictions are apparently not unequivocal, and there is experimental evidence showing that the purified ComQ protein is enzymatically active *in vitro* even in the absence of a membrane [27]. Therefore the condition for ComQ being a membrane protein was not included in our survey criteria. It is not known how ComX, once post-translationally modified, leaves the cell. Nevertheless, the expression of ComX and ComQ in *E. coli* is sufficient to reconstitute the synthesis of an active ComX [7].

### ComX

In all *B. subtilis* strains known thus far, the *comX* gene codes for a 52 to 73 residue-long precursor protein. The sequence of ComX is extremely variable (PFAM identifier: PF05952), but it contains a tryptophan within the 5 to 10 amino acids of the C terminus [18].

### ComP

**Figure 1:**
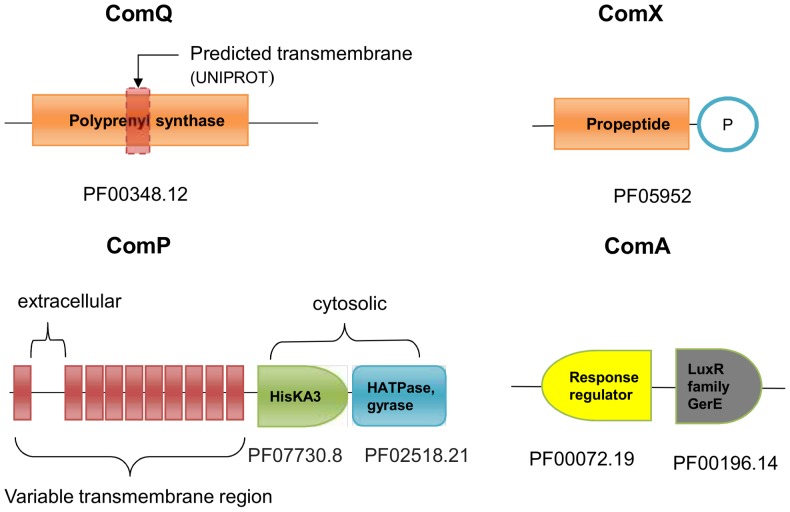
Domain structure of the Com proteins. The codes denote the PFAM (PF) domain identifiers.

Within the receptor ComP the two conserved intracellular motifs were included into the HMM model: histidine kinase domain (PFAM: PF07730) and an ATP-binding domain (PFAM: PF02518). The rest of the protein consists of a series of transmembrane helices separated by intra and extracellular loops, (**Figure 1**, also see the section on non-*B. subtilis*-like loci, below). The ComX binding site is located on the extracellular part of its N-terminal membrane-associated domain [6].

### ComA

ComA is polypeptide of 196 to 245 residues in length (average length 214 residues). It has a typical structure of the transcriptional response regulator. The N-terminal domain (PFAM: PF00072.19) carries the phosphorylation domain involving an Asp residue. The C-terminal DNA-binding domain (PFAM: PF00196.14) is responsible for DNA binding [28], [29].

In this work we searched bacterial genomes for candidate loci similar to the *B. subtilis* ComQXPA locus, by combining Hidden Markov Model (HMM) recognizers with filtering criteria based on the structural and organizational properties of ComQXPA QS systems. Here we show that the *comQXPA*-like gene arrangements are present in Firmicutes, outside the *B. subtilis* species. The candidate loci are characterized by similar sequence diversity profiles, but notable differences were also revealed in the overlap patterns found between *comQXPA* genes.

## Results and Discussion

### Census of *comQXPA* loci in prokaryotic genomes

The complex nature of the ComQXPA quorum sensing system justifies questioning whether or not related circuit architectures occur outside the *B. subtilis* species. We tried to answer this question by scanning all prokaryotic genomes (2620 complete and 6970 draft genomes having 644474 annotated and 505155 unannotated contigs, and a total of over 4.7 million protein sequences) for ComQXPA-related proteins, using Hidden Markov recognizers and additional filtering as described in Methods section. This census revealed that in addition to the 21 occurrences explicitly mentioned in the literature and/or in the databases, there are 39 new occurrences in which one, more or all of the functions were indicated as hypothetical. The complete list of the species is shown in **Table S1**.

**Figure 2:**
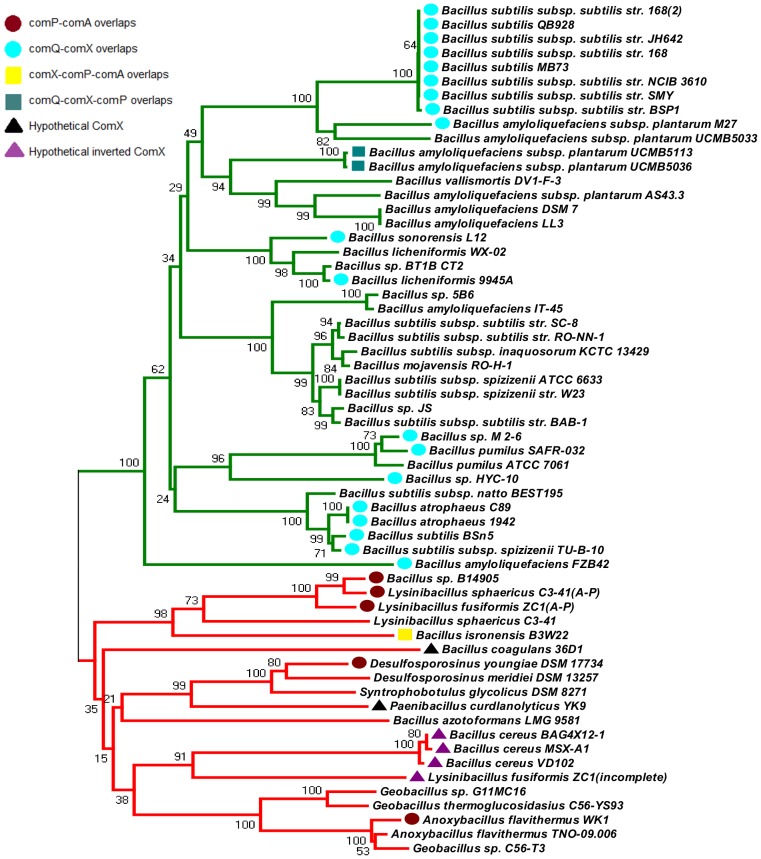
A similarity cladogram of ComQ sequences. The larger clade (green) that we termed “*B. subtilis*-like” contains the *B. subtilis* and a few other species from the *Bacillus* genus that are closely related. The other, visibly more varied one (red) contains non-*B. subtilis* sequences. The overlaps are indicated as highlighted bullets before each species.

Among the species in Table S1 we find a number of occurrences in which Com proteins have not yet been described. The similarity cladograms of all four ComQXPA sequences showed two large clades. **Figure 2** shows the ComQ tree as an example, while the trees of ComX, ComP and ComA are shown in Figures S1–S3. The larger clade, which we termed “*B. subtilis*-like”, contains *B. subtilis* and a few other species from the *Bacillus subtilis-lichenoformis* group. We termed the other, visibly more varied clade as “non-*B. subtilis*-like” since it contains no *B. subtilis* sequences. This clade contains a few species from the *Bacillus* genus (*B. isronensis, B. coagulans, B. azotoformansname and B. cereus*), but also species from *Lysinbacillus, Geobacillus, Anoxybacillus, Desulfosporosinus* and more distantly related organisms such as *Clostridiales*. (Note that the same clades appear on the ComP and ComA trees, shown in Figures S2 and S3). To our knowledge, *comQXPA*- like loci in the members of this clade have not been reported before, so we carried out a detailed comparison of the two clades in terms of gene arrangements and sequence diversity.

### Local arrangement of the *comQXPA* genes

**Figure 3:**
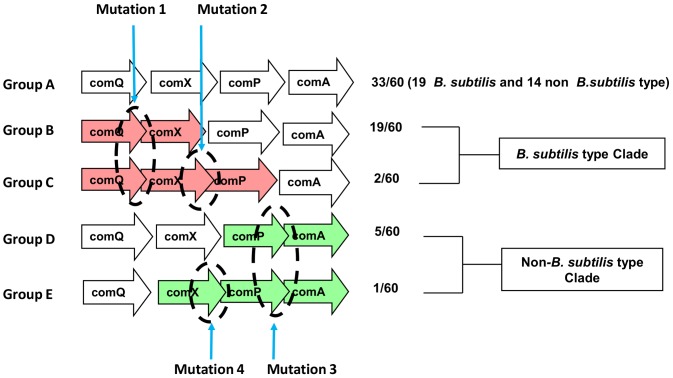
The gene overlaps within the comQXPA loci in *B. subtilis* type and non- *B. subtilis* type clade, overlaps are shown in color. For the definition of the clades, see phylogenetic analysis. The numbers indicate the frequency of the type/the number total occurrences (i.e. 60).

The chromosomal arrangements of the loci are schematically shown in **Figure 3** where the reading frames are represented as arrows pointing to the putative direction of transcription. The types of overlaps were named A through E, and the species are given in Table S1. The majority of the loci contain no overlapping reading frames (A in Figure 3). A substantial number of the found loci contain overlapping reading frames that are spread in the two clades. In the *B. subtilis*-like clade the overlap types are dominated by an apparent mutation of the *comQ* stop codon. This results in a 13–18 amino acids long C-terminal extension, giving rise to the *comQ-comX* overlaps. In two loci within the same clade (*B. amyloliquefaciens subsp. plantarum* UCMB5113, *B. amyloliquefaciens subsp. plantarum* UCMB5036), there is a second mutation causing a 14 amino acids long N-terminal extension to ComP, leading to the comQ-comX-comP overlaps. ii) In the non-*B. subtilis*-like clade the most frequent overlaps (six out of 14 species in the clade) are between ComP and ComA of which five are caused by a C-terminal extension of ComP. In contrast, in *Anoxybacillus flavithermus* WK1 the overlap is due to a 22 amino acids long N-terminal extension of ComA. In one locus (*B. isronensis* B3W22) there is an additional mutation that gives rise to a three amino acids long N-terminal extension of ComP, leading to a *comX-comP-comA* overlap.

In general, overlapping reading frames are not rare in bacteria [30] most likely because bacterial genes are frequently (>70%) located on one strand [31]. However, the fact that we see two coherent sets of mutations (Mutations 1–2, and Mutations 3–4, respectively) that are confined to two separate clades makes us believe that these mutations may have some logical reason for existing. In fact, the estimated probability of finding these mutations in two separate groups by chance is low. More precisely, a clear differentiation in the overlap type groups is evident, as both F-tests and P-tests [32] are highly significant (for F -test: p<0.0002; for P-test: p<0.001) which is further supported by the high bootstrap values obtained between the clades (Figure 2 and Figures S1 and S2). It has been suggested that overlapping reading frames may result in more efficient transcriptional control and reduce the need for more complex regulatory pathways [30]. Similarly, overlapping genes are often found in regulatory operons and indeed, the primary role of *comQXPA* loci is to control gene expression [5]. We thus speculate that the expression of *comQXPA* genes may be different in the two clades. In other words, the fact that different kinds of mutations are accepted in the two clades makes us hypothesize that the *com* loci of two clades may differ in terms of how the transcription/translation of the genes is coupled – a statement that would be worth testing by experimental methods in the future.

### Unusual com-like loci

**Figure 4:**
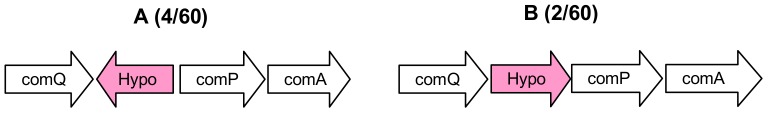
Unusual comQXPA-like loci. (A) Non-canonical unusual *com* system is present in *B. cereus* VD102, *B. cereus* BAG4X12 1, *B. cereus* MSX A1 and *L. fusiformis* ZC1. Note that *Lysinibacillus* is the only genus whose two species have two *com* loci each: *L. sphaericus* C3 41 has two canonical loci while in *L. fusiformis* ZC1 one locus is canonical and the other one is non-canonical, shown here. (B) Canonical unusual *com* system present in *Paenibacillus curdlanolyticus* YK9 and *B. coagulans* 36D1.

A few *com*-like arrangements were found outside the *Bacillu*s genus in which the *comX* sequence was of the right length and in the right position between *comQ* and *comP*, but showed no appreciable homology with the known *comX* sequences, except the tryptophan residue in the C-terminus. We term these reading frames as hypothetical *comX*, (**Figure 4**). In one of the groups the hypothetical *comX* gene is on the opposite strand (4A). Three strains (*B. cereus* VD102, *B. cereus* BAG4X12 1, *B. cereus* MSX A1) and *L. fusiformis* ZC1 have this arrangement. We note that *Lysinibacillus* is the only genus whose two species have two *comQXPA* loci each: *L. sphaericus C3 41* has two canonical loci while in *L. fusiformis* ZC1 one locus is canonical and the other has the hypothetical *comX* gene on the opposite strand, shown in Figure 4A.

The other arrangement (Figure 4B) is canonical in terms of the serial order of the genes. Two species *Paenibacillus curdlanolyticus* YK9 and *B. coagulans* 36D1 have this kind of arrangement. We note that we have no reason to believe that these *com*-like loci are functional or that they function in the same way as in *B. subtilis*. Nevertheless, the fact that they are at least in part conserved in relatively distinct species makes them interesting subjects for further experiments.

### Sequence variability

As the non-*B. subtilis*-like clade contained interesting and novel *comQXPA* loci, which are known to be highly polymorphic within *B. subtilis* species [7], [18], [21], [23], [24], we compared the variability of the sequences within this clade and the more established *B. subtilis*-like clade.

The variability (π) of the ComQXPA proteins (Table 1) was calculated as the number of amino acid differences per site and by averaging over all sequence pairs using the MEGA program [33]. The non-*B. subtilis*-like clade was consistently more diverse than the *B. subtilis*-like which is in accordance with the branch lengths of the cladograms (see **Figure 2** and Figures S1–S3). In both clades the conservation of the genes followed the same order: ComA>ComP>ComQ>ComX. ComX showed the highest within-clade variability. Moreover, the diversity of the *com* sequences is substantially above the variability of the example housekeeping gene product RpoB (Table 1).

**Table 1:** Sequence diversity^1^ of ComQXPA proteins.

	*B. subtilis* clade	Non-*B.subtilis* clade	Clades combined
**ComA**	0.13±0.01	0.57±0.02	0.41±0.02
**ComP**	0.29±0.01	0.64±0.01	0.51±0.01
**ComQ**	0.46±0.02	0.60±0.01	0.57±0.02
**ComX**	0.63±0.04	0.69±0.03	0.70±0.03
**RpoB** 2	0.017±0.002	0.15±0.01	0.091±0.005

1 π diversity values calculated with the MEGA program [33] from the multiple alignments used to construct the cladograms. High values indicate high diversity.

2 RNA polymerase beta subunit, taken as a example of a conserved housekeeping protein.

**Figure 5:**
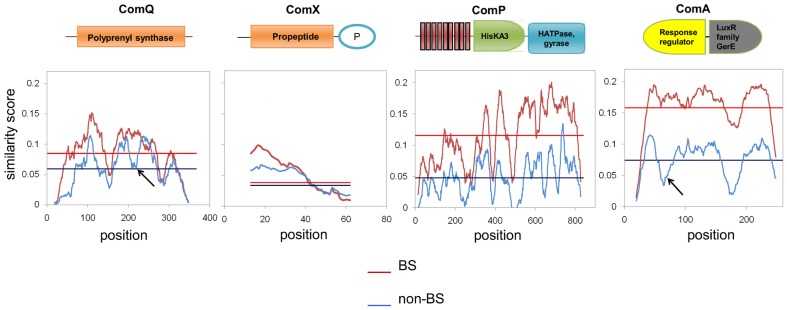
Variability of Com protein sequences in the *B. subtilis*-like (red) and non-*B. subtilis*-like (blue) proteins. The average variability of the two clades is plotted for each protein respectively [32], the straight lines represent the average value of the plots. Note that the overall course of the variability is similar in both clades, i.e. peaks and valley are at the same position, and the non-*B. subtilis*-like group are more variable than the *B. subtilis*-like proteins. A conspicuous difference between the two clades is found in ComA protein where the response regulator domain in the non-*B. subtilis*-like clade has a variable region (valley indicated by arrow).

The distribution of amino acid variability along the protein sequences was investigated by calculating the similarity scores for each position using the Plotcon program [34]. Here, high values indicate more conserved regions (**Figure 5**). It is conspicuous that the general course of the variability curves is similar between the two clades, i.e. the peaks and the valleys are at similar sequence positions, which further supports the suggestion that the proteins in both clades are homologous and are under broadly similar evolutionary constraints. It is also conspicuous that the proteins of the non-*B. subtilis*-like clade are reasonably more variable than their counterparts in the *B.subtilis*-like clade, i.e. the blue curves run under the red curves. In other words, we see the same general tendency as in Table 1. The highest variability is seen at the C terminus of ComX that encodes the active peptide signal, whereas the rest of the propeptide is apparently more conserved. This pattern is evident for both clades.

A substantial variability was also detected in the N-terminal domain of the ComP protein that is predicted to interact with ComX [6]. The variability of the N-terminal domain is especially pronounced in comparison to the conserved cytoplasmic domain. Although earlier studies suggested that the N-terminal domain of ComP contains 8 transmembrane helices [6], the prediction methods used in this work suggest that the N-terminal domain contains 10 transmembrane helices (48 out of 60 occurrences). However, there are proteins in both clades in which the N-terminal transmembrane domain is missing (9 out of 60 occurrences, uniformly distributed in the two clades) meaning that the loop region of the intact protein becomes N-terminal (Table S3). Another interesting example of sequence variability is an unusual, C-terminal extension of ComX, that occurs only in the B. subtilis-like clade (*B. subtilis* natto, *B. subtilis* subsp. Spizizenii TU-B-10, *B. subtilis* BSn5, *Bacillus atrophaeus* 1942 and *Bacillus atrophaeus* C89)

Even though the residue conservation follows the same general trends in both clades, there are a few interesting points that need to be discussed. It is not known which part in ComQ is responsible for the specificity of interactions between ComQ and ComX. However, we speculate that the two valleys (positioned at around 150 and 280, respectively) indicating a high degree of polymorphism might be responsible for these interactions. Although this prediction is highly speculative it should be interesting to address this problem by experimental methods in the future. ComA shows lowest overall diversity and there is one noteworthy difference between the plots of ComA proteins. The region of high variability is present around position 80 in the non-*B. subtilis* like clade, but it is absent in the *B. subtilis* like clade. The function of this variable region is not known but we speculate that it might be involved in interactions with additional, as yet unidentified modulators. Again, we point to this region as it might be of interest for future experimental studies.

### Common traits of ComQXPA and AHL based QS systems

It is interesting to compare the general features of *comQXPA* loci to the AHL regulatory system of Gram negative bacteria [35]–[37]. At first sight, the AHL system is very different in many respects. Firstly, its signal molecules, *N*-acyl homoserine lactones, are secondary metabolites, as opposed to the post-translationally modified, ribosomally synthesized ComX. Secondly, the core AHL system is simpler as it contains only two proteins, the signal synthetase and a LuxR-like response regulator. Most AHL systems contain well-defined additional negative feedback regulatory components (co-expressed repressors, RNA interference etc. [35], [37]). Recently, it was shown that inhibition is also part of the ComQXPA locus; ComQ was found to provide negative feedback that modulates the QS response of the signal producer [19]. In addition, the overall architecture of the com-specific response regulator ComA and all AHL LuxR proteins bear similarities in as much as the C-terminal DNA-binding domain belongs to the same domain type (LuxR family GerE), and the signal-mediating domain is at the N-terminal in both proteins. The underlying signaling mechanisms are naturally different since LuxR binds the AHL autoinducer molecule while the N-terminal domain of ComA is phosphorylated by the histidine kinase receptor ComP. Both systems contain conserved gene overlaps (40% of the systems in AHL and 45% in com In terms of sequence similarity, AHL proteins in different topological arrangements are typical orthologs that are closer to their QS-linked homologs in other species [35]–[37], than to the related proteins in the same genome. The same tendency holds for the Com proteins, even though the analysis could only be done for ComP proteins which have clear-cut non-QS homologues in most bacterial genomes. Here we see a similar tendency (Figure S4 and explanations in figure legend): QS-linked ComP proteins cluster together with other ComPs from different genomes rather than clustering with other histidine kinases of the same genome. This shows that ComP has diverged from the other, non-QS linked histidine kinases before the modern bacterial species appeared, i.e. the emergence of ComQXPA QS systems is a not a recent evolutionary event.

## Conclusions

In this work, we presented a census of *comQXPA* locus proteins involved in the quorum sensing regulation of competence and other late growth adaptive traits in *B. subtilis*. We found 31 new occurrences, many of them outside the *Bacillus* genus, with some from different orders (for instance order: Clostridiales, family: *Peptococcaceae*, genera: *Desulfosporosinus, Syntrophobotulus*). The local arrangement of the genes was quite conserved in all *com*-like occurrences. The only variability of gene topology was found in putative *com*-related operons whose function may however, not be necessarily linked to quorum sensing, or may be based on a different type of peptide signal. We found two conserved classes of overlap patterns in *B. subtilis*-like and non-*B subtilis* like *comQXPA* loci, respectively, which may be due to hitherto unknown transcriptional/translational differences.

In summary, a wholesale scan of current databases showed a number of novel occurrences of com QS regulatory locus originally identified in *B. subtilis*. It was found that this locus is phylogenetically more widespread than previously thought, and its organization has some commonalities with unrelated QS systems such as conserved gene overlaps.

## Methods

### Data

Data relating to 2620 complete genomes and 6970 drafts genomes having 644474 annotated and 505155 unannotated contigs were downloaded from NCBI FTP, ftp://ftp.ncbi.nlm.nih.gov/genomes/Bacteria/ and ftp://ftp.ncbi.nlm.nih.gov/genomes/Bacteria_DRAFT respectively. Additional protein sequences were retrieved from the UNIPROT database (http://www.uniprot.org/). Data retrieval was completed on August 16, 2013.

### QS gene detection

*com* genes were determined using Hidden Markov Model (HMM) recognizers built with HMMER 3.0, http://hmmer.janelia.org/). Briefly, a core set of protein sequences was taken for ComA, ComP, ComX and ComQ sequences from Uniprot database (see Table S2). The CLUSTAL program (accessed via the EBI Webportal, http://www.ebi.ac.uk/Tools/msa/clustalw2/) was used for constructing the multiple sequence alignments which were then processed by the HMMBBUILD program to give HMM recognizers that were in turn used to scan the protein sequence data (over 4.7 million protein sequences). Sequences giving an E-value below 0.1 were manually checked for length, alignment coverage and residue conservation. It was also determined whether the loci were full or incomplete. We proceeded with the following steps:

1.) The presence of transmembrane helices in ComP candidates was checked by TMHMM server v. 2.0 (http://www.cbs.dtu.dk/services/TMHMM/).

2.) The presence of a tryptophan residue at the end of each probable ComX was manually checked.

3.) Only those proteins were accepted where the length was within the range of protein sequences used for building the HMM recognizers. The hypothetical proteins in place of ComX also fell within this range.

Cladograms of ComA, ComP, ComX and ComQ sequence groups were built using the guide tree of the CLUSTAL program and visualized using the MEGA 5 program package installed from http://www.megasoftware.net. In the trees, the numerical value at each node indicates the bootstrap value supporting every split in the lineage (out of 1,000 bootstrap replicates).

The complete list of the species having *com* loci is shown in **Table S1**.

### Statistical analysis

For the distribution of overlaps, the P-test of significance [32] was performed using MacClade 4.08 [38]. The P-test seeks for the co-variation of the sequence phylogeny with the distribution of the overlap-types among the groups. One way to study this is to use parsimony [39], which provides the minimum number of changes (i.e. switch from one type of overlap to another) necessary to explain the observed distribution of sequences among the groups, assuming that the group type (overlap type) evolved according to the sequence based phylogenetic tree [32]. The minimal number of changes between a given phylogeny and random phylogenies can be determined and significance values can then be calculated. F-test was performed on ComQ amino acids sequences as described by Martin [32]. Statistical significance was evaluated by randomly assigning sequences to group types and calculating F-values for 10000 permutations. The F-test assesses the degree of differentiation between the groups by comparing the total genetic diversity within each group to the total genetic diversity of the groups combined [40]. If the members within the groups (overlap type) are genetically very similar, but the groups themselves are very different from each other, the F value will be significantly higher than zero, which is an indicator of a correlation between the genetic sequences and the group type (i.e. overlap type) [41]. Other statistical tests were carried out as described in [42], [43].

## Supporting Information

Figure S1 A similarity cladogram of ComA sequences.
(TIF)

Figure S2 A similarity cladogram of ComP sequences.
(TIF)

Figure S3 A similarity cladogram of ComX sequences.
(TIF)

Figure S4 Cladogram of ComP and other Histidine Kinase protein sequences in 60 genomes in which *comQXPA* locus was identified.
(TIF)

Table S1 List of the species having *comQXPA* loci.
(DOC)

Table S2 List of protein sequence sets used for building HMM recognizers.
(DOCX)

Table S3 Transmembrane domain architecture in ComP proteins.
(DOC)
