# Supplementary figures and images for: ComQXPA Quorum Sensing Systems May Not Be Unique to Bacillus subtilis: A Census in Prokaryotic Genomes
Source: PLoS One. 2014 May 2;9(5):e96122. doi: 10.1371/journal.pone.0096122 (PMC4008528; doi:10.1371/journal.pone.0096122)

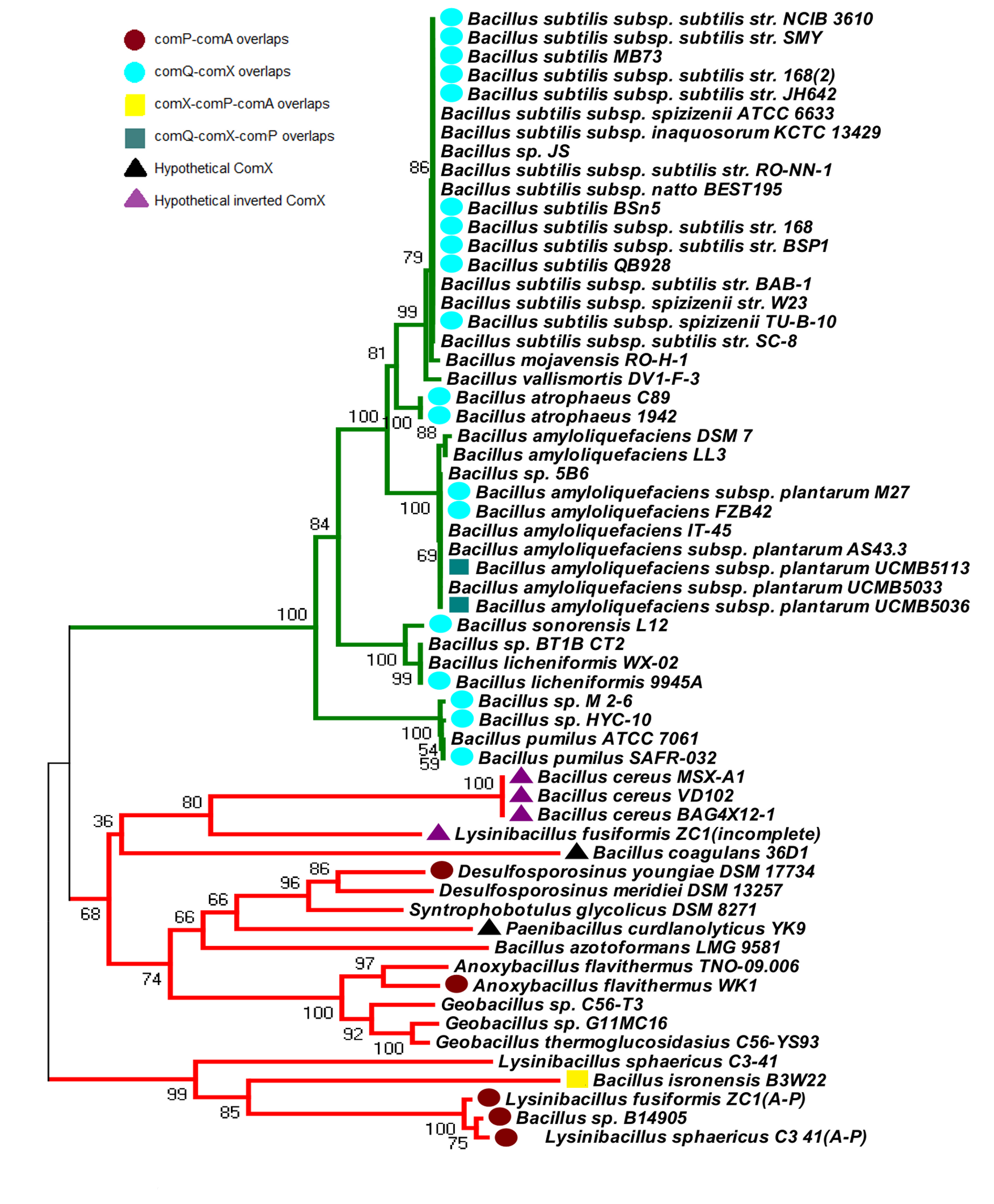

Supplement: Figure S1 — A similarity cladogram of ComA sequences. (TIF) [file pone.0096122.s001.tif]

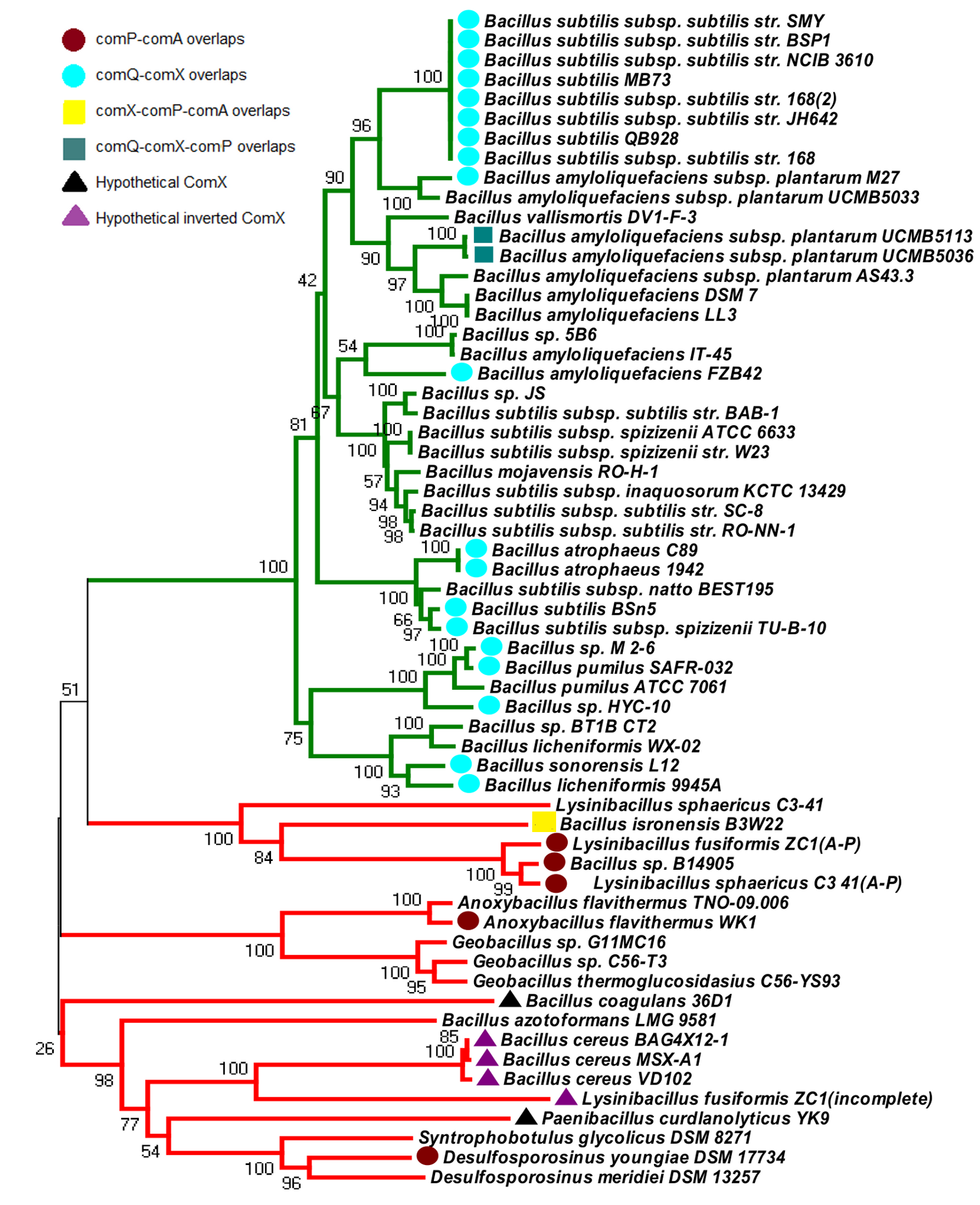

Supplement: Figure S2 — A similarity cladogram of ComP sequences. (TIF) [file pone.0096122.s002.tif]

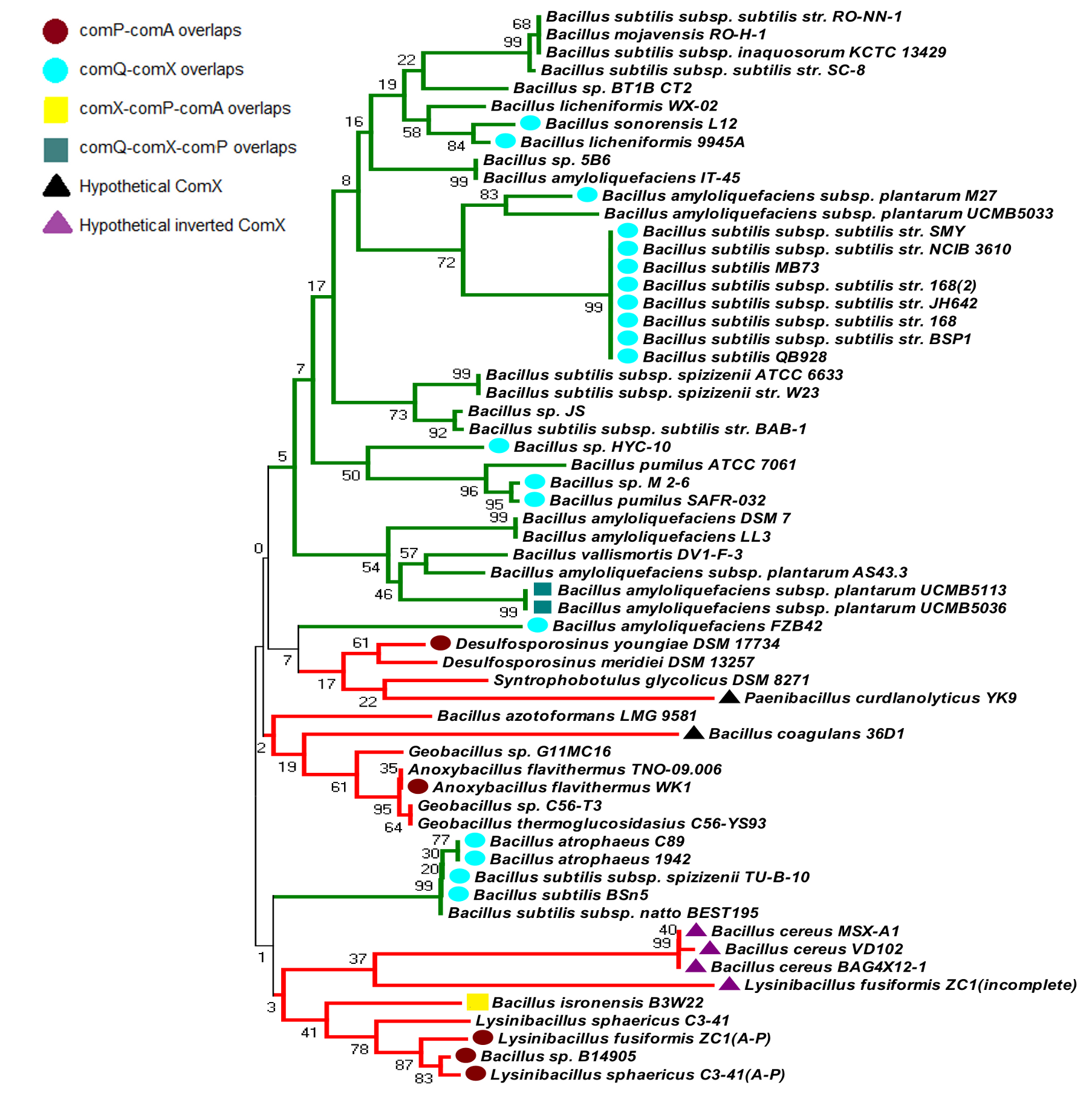

Supplement: Figure S3 — A similarity cladogram of ComX sequences. (TIF) [file pone.0096122.s003.tif]

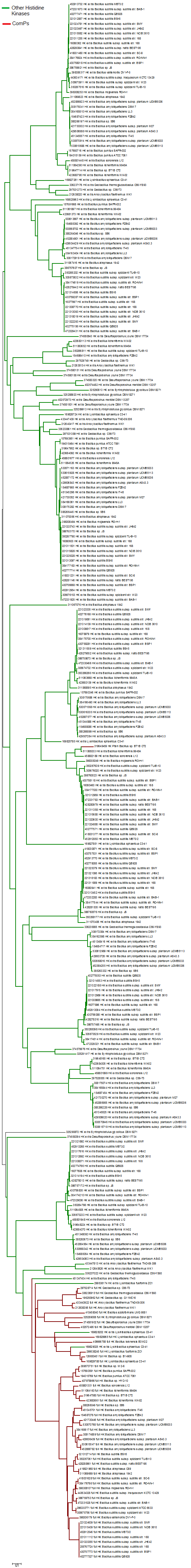

Supplement: Figure S4 — Cladogram of ComP and other Histidine Kinase protein sequences in 60 genomes in which comQXPA locus was identified. (TIF) [file pone.0096122.s004.tif]
