# Supplementary material for: ComQXPA Quorum Sensing Systems May Not Be Unique to Bacillus subtilis: A Census in Prokaryotic Genomes
Source: PLoS One. 2014 May 2;9(5):e96122. doi: 10.1371/journal.pone.0096122 (PMC4008528; doi:10.1371/journal.pone.0096122)
Supplement: Table S1 — List of the species having comQXPA loci. (DOC) [file pone.0096122.s005.doc]

**Table S1: List of genomes in which *comQXPA-* like proteins were identified**

| **Genome name**  **Chromosome/contig)** | **GI** | **Strand** | **From** | **To** | **Our annotation** | | | **Ref^3^** |
| --- | --- | --- | --- | --- | --- | --- | --- | --- |
|  |  |  |  |  | **Function** | **Clade^1^** | **Overlap type^2^** |  |
| **Bacillus HYC 10** | 407978965 | - | 16598 | 17239 | ComA | S |  |  |
|  | 407978966 | - | 17320 | 19641 | ComP | S |  |  |
| NZ_AMSH01000014 | 407978967 | - | 19695 | 19865 | ComX | S | B |  |
|  | 407978968 | - | 19862 | 20776 | ComQ | S | B |  |
|  |  |  |  |  |  |  |  |  |
| **Bacillus M 26** | 389573750 | - | 45674 | 46315 | ComA | S |  |  |
|  | 389573751 | - | 46396 | 48561 | ComP | S |  |  |
| NZ_AJWW01000025 | 389573752 | - | 48716 | 48886 | ComX | S | B |  |
|  | 389573753 | - | 48864 | 49787 | ComQ | S | B |  |
|  |  |  |  |  |  |  |  |  |
| **Bacillus 5B6** | 385266048 | - | 2805760 | 2806404 | ComA | S | A |  |
|  | 385266049 | - | 2806485 | 2808773 | ComP | S | A |  |
| NZ_AJST01000001 | 385266050 | - | 2808785 | 2808949 | ComX | S | A |  |
|  | 385266051 | - | 2808949 | 2809860 | ComQ | S | A |  |
|  |  |  |  |  |  |  |  |  |
| **Bacillus B14905** | 126650450 | - | 5998 | 6618 | ComA | N | D |  |
|  | 126650451 | - | 6593 | 8839 | ComP | N | D |  |
| NZ_AAXV01000005 | 126650452 | - | 8887 | 9045 | ComX | N |  |  |
|  | 126650453 | - | 9060 | 9953 | ComQ | N |  |  |
|  |  |  |  |  |  |  |  |  |
| **Bacillus BT1B CT2** | 319647579 | - | 403211 | 403849 | ComA | S | A |  |
|  | 319647580 | - | 403936 | 406236 | ComP | S | A |  |
| NZ_GL635727 | 319647581 | - | 406238 | 406411 | ComX | S | A |  |
|  | 319647582 | - | 406415 | 407296 | ComQ | S | A |  |
|  |  |  |  |  |  |  |  |  |
| **B. subtilis_SMY** | 221324638 | - | 3251855 | 3252499 | ComA | S |  | [1–5] ^4^ |
|  | 221324639 | - | 3252580 | 3254889 | ComP | S |  |  |
| NZ_CM000490 | 221324640 | - | 3254904 | 3255071 | ComX | S | B |  |
|  | 221324641 | - | 3255059 | 3255958 | ComQ | S | B |  |
|  |  |  |  |  |  |  |  |  |
| ***B. subtilis* NCIB_3610** | 221315438 | - | 3251815 | 3252459 | ComA | S |  | [1–5] ^4^ |
|  | 221315439 | - | 3252540 | 3254849 | ComP | S |  |  |
| NZ_CM000488 | 221315440 | - | 3254864 | 3255031 | ComX | S | B |  |
|  | 221315441 | - | 3255019 | 3255918 | ComQ | S | B |  |
|  |  |  |  |  |  |  |  |  |
| ***B. subtilis* MB732** | 452913019 | - | 74262 | 74906 | ComA | S |  | [1–5] ^4^ |
|  | 452912546 | - | 74987 | 77296 | ComP | S |  |  |
| NZ_AOTY01000001 | 452912502 | - | 77311 | 77478 | ComX | S | B |  |
|  | 452912646 | - | 77466 | 78365 | ComQ | S | B |  |
|  |  |  |  |  |  |  |  |  |
| ***B. subtilis* 168** | 221311111 | - | 3251761 | 3252405 | ComA | S |  | [2] |
|  | 221311112 | - | 3252486 | 3254795 | ComP | S |  |  |
| NZ_CM000487 | 221311113 | - | 3254810 | 3254977 | ComX | S | B |  |
|  | 221311114 | - | 3254965 | 3255864 | ComQ | S | B |  |
|  |  |  |  |  |  |  |  |  |
| ***B. subtilis* SC_8** | 418031626 | + | 1041642 | 1042586 | ComQ | S | A | [1–5] ^4^ |
|  | 418031627 | + | 1042601 | 1042762 | ComX | S | A |  |
| NZ_AGFW01000001 | 418031628 | + | 1042860 | 1045067 | ComP | S | A |  |
|  | 418031629 | + | 1045127 | 1045792 | ComA | S | A |  |
|  |  |  |  |  |  |  |  |  |
| ***B. subtilis* JH642** | 221320354 | - | 3224835 | 3225479 | ComA | S |  | [1] |
|  | 221320355 | - | 3225560 | 3227869 | ComP | S |  |  |
| NZ_CM000489 | 221320356 | - | 3227884 | 3228051 | ComX | S | B |  |
|  | 221320357 | - | 3228039 | 3228938 | ComQ | S | B |  |
|  |  |  |  |  |  |  |  |  |
| ***B. subtilis* spizizenii ATCC 6633** | 296330769 | + | 47108 | 47968 | ComQ | S | A | [3]^4^ |
|  | 296330770 | + | 47981 | 48145 | ComX | S | A |  |
| NZ_ADGS01000012 | 296330771 | + | 48153 | 50477 | ComP | S | A |  |
|  | 296330772 | + | 50558 | 51202 | ComA | S | A |  |
|  |  |  |  |  |  |  |  |  |
| ***B. subtilis* inaquosorum KCTC 13429** | 443634326 | + | 189370 | 190347 | ComQ | S | A | [3]^4^ |
|  | 443634327 | + | 190362 | 190523 | ComX | S | A |  |
| NZ_AMXN01000007 | 443634328 | + | 190693 | 192828 | ComP | S | A |  |
|  | 443634329 | + | 192909 | 193553 | ComA | S | A |  |
|  |  |  |  |  |  |  |  |  |
| ***B. cereus* VD102** | 423608524 | - | 462923 | 463576 | ComA | N | A |  |
|  | 423608525 | - | 463582 | 465897 | ComP | N | A |  |
| NZ_JH792240 | 423608526 | + | 466014 | 466181 | - | N | A |  |
|  | 423608527 | - | 466183 | 467112 | ComQ | N | A |  |
|  |  |  |  |  |  |  |  |  |
| ***B. cereus* BAG4X12_1** | 423438530 | + | 4689952 | 4690881 | ComQ | N | A |  |
|  | 423438531 | - | 4690883 | 4691050 | - | N | A |  |
| NZ_JH791951 | 423438532 | + | 4691167 | 4693482 | ComP | N | A |  |
|  | 423438533 | + | 4693488 | 4694141 | ComA | N | A |  |
|  |  |  |  |  |  |  |  |  |
| ***B. cereus* MSX_A1** | 423565994 | - | 452254 | 452907 | ComA | N | A |  |
|  | 423565995 | - | 452913 | 455228 | ComP | N | A |  |
| NZ_JH792105 | 423565996 | + | 455345 | 455512 | - | N | A |  |
|  | 423565997 | - | 455514 | 456443 | ComQ | N | A |  |
|  |  |  |  |  |  |  |  |  |
| ***B. isronensis* B3W22** | 406666784 | - | 30729 | 31346 | ComA | N | E |  |
|  | 406666785 | - | 31321 | 33618 | ComP | N | E |  |
| NZ_AMCK01000012 | 406666786 | - | 33602 | 33760 | ComX | N | E |  |
|  | 406666787 | - | 33771 | 34664 | ComQ | N |  |  |
|  |  |  |  |  |  |  |  |  |
| ***B. sonorensis* L12** | 458801300 | - | 41264 | 41902 | ComA | S |  |  |
|  | 458801301 | - | 41984 | 44296 | ComP | S |  |  |
| NZ_AOFM01000009 | 458801302 | - | 44311 | 44484 | ComX | S | B |  |
|  | 458801303 | - | 44468 | 45367 | ComQ | S | B |  |
|  |  |  |  |  |  |  |  |  |
| ***B. pumilus* ATCC 7061** | 194015634 | - | 211408 | 212049 | ComA | S | A |  |
|  | 194015769 | - | 212130 | 214421 | ComP | S | A |  |
| NZ_ABRX01000002 | 194015143 | - | 214428 | 214577 | ComX | S | A |  |
|  | 194015360 | - | 214585 | 215508 | ComQ | S | A |  |
|  |  |  |  |  |  |  |  |  |
| ***B. licheniformis* WX 02** | 423683660 | - | 3362792 | 3363430 | ComA | S | A | [6]^5^, [7,8]^4^ |
|  | 423683661 | - | 3363517 | 3365823 | ComP | S | A |  |
| NZ_JH636050 | 423683662 | - | 3365846 | 3366010 | ComX | S | A |  |
|  | 423683663 | - | 3366019 | 3366900 | ComQ | S | A |  |
|  |  |  |  |  |  |  |  |  |
| ***B. amyloliquefaciens plantarum* M27** | 421730444 | + | 732759 | 733724 | ComQ | S | B |  |
|  | 421730445 | + | 733687 | 733857 | ComX | S | B |  |
| NZ_AMPK01000004 | 421730446 | + | 733877 | 736180 | ComP | S |  |  |
|  | 421730447 | + | 736261 | 736905 | ComA | S |  |  |
|  |  |  |  |  |  |  |  |  |
| ***B. atrophaeus* C89** | 419821966 | + | 32850 | 33719 | ComQ | S | B |  |
|  | 419821967 | + | 33716 | 33937 | ComX | S | B |  |
| NZ_AJRJ01000035 | 419821968 | + | 33953 | 36262 | ComP | S |  |  |
|  | 419821969 | + | 36344 | 36985 | ComA | S |  |  |
|  |  |  |  |  |  |  |  |  |
| ***B. azotoformans* LMG 9581** | 410458039 | - | 2395 | 3060 | ComA | N | A |  |
|  | 410458040 | - | 3076 | 5415 | ComP | N | A |  |
| NZ_AJLR01000035 | 410458041 | - | 5509 | 5670 | ComX | N | A |  |
|  | 410458042 | - | 5711 | 6619 | ComQ | N | A |  |
|  |  |  |  |  |  |  |  |  |
| ***B. mojavensis* RO H 1** | 398308106 | - | 76612 | 77256 | ComA | S | A | [4] |
|  | 398308107 | - | 77337 | 79634 | ComP | S | A |  |
| NZ_JH600279 | 398308108 | - | 79642 | 79803 | ComX | S | A |  |
|  | 398308109 | - | 79817 | 80677 | ComQ | S | A |  |
|  |  |  |  |  |  |  |  |  |
| ***B. vallismortis* DV1 F 3** | 398306178 | - | 112511 | 113155 | ComA | S | A | [3]^5^ |
|  | 398306179 | - | 113236 | 115554 | ComP | S | A |  |
| NZ_JH600244 | 398306180 | - | 115574 | 115750 | ComX | S | A |  |
|  | 398306181 | - | 115765 | 116637 | ComQ | S | A |  |
|  |  |  |  |  |  |  |  |  |
| ***Geobacillus* G11MC16** | 196250961 | - | 15553 | 16224 | ComA | N | A |  |
|  | 196250962 | - | 16250 | 18514 | ComP | N | A |  |
| NZ_ABVH01000020 | 196250963 | - | 18521 | 18676 | ComX | N | A |  |
|  | 196250964 | - | 18694 | 19626 | ComQ | N | A |  |
|  |  |  |  |  |  |  |  |  |
| ***Anoxybacillus flavithermus* TNO_09_006** | 433443920 | + | 581038 | 581928 | ComQ | N | A |  |
|  | 433443921 | + | 581950 | 582105 | ComX | N | A |  |
| NZ_KB205935 | 433443922 | + | 582115 | 584370 | ComP | N | A |  |
|  | 433443923 | + | 584397 | 585047 | ComA | N | A |  |
|  |  |  |  |  |  |  |  |  |
| ***Lysinibacillus fusiformis* ZC1** | 299538173 | - | 105302 | 105937 | ComA | N | A |  |
|  | 299538174 | - | 105943 | 108228 | ComP | N | A |  |
| NZ_ADJR01000053 | 299538175 | + | 108294 | 108467 | - | N | A |  |
|  | 299538176 | - | 108471 | 109400 | ComQ | N | A |  |
|  |  |  |  |  |  |  |  |  |
| ***Lysinibacillus fusiformis* ZC1** | 299538243 | + | 179719 | 180621 | ComQ | N |  |  |
|  | 299538244 | + | 180634 | 180792 | ComX | N |  |  |
| NZ_ADJR01000053 | 299538245 | + | 180810 | 183086 | ComP | N | D |  |
|  | 299538246 | + | 183061 | 183681 | ComA | N | D |  |
|  |  |  |  |  |  |  |  |  |
| ***Paenibacillus curdlanolyticus* YK9** | 304408345 | - | 19156 | 19824 | ComA | N | A |  |
|  | 304408346 | - | 19840 | 22224 | ComP | N | A |  |
| NZ_AEDD01000016 | 304408347 | - | 22349 | 22513 | - | N | A |  |
|  | 304408348 | - | 22660 | 23592 | ComQ | N | A |  |
|  |  |  |  |  |  |  |  |  |
| ***Desulfosporosinus youngiae* DSM 17734** | 374581901 | - | 3509306 | 3509971 | ComA | N | D |  |
|  | 374581902 | - | 3509968 | 3512334 | ComP | N | D |  |
| NZ_CM001441 | 374581903 | - | 3512389 | 3512556 | ComX | N |  |  |
|  | 374581904 | - | 3512666 | 3513580 | ComQ | N |  |  |
|  |  |  |  |  |  |  |  |  |
| **Bacillus_JS** | 386759752 | - | 3117558 | 3118202 | ComA | S | A |  |
|  | 386759753 | - | 3118283 | 3120583 | ComP | S | A |  |
| NC_017743 | 386759754 | - | 3120595 | 3120759 | ComX | S | A |  |
|  | 386759755 | - | 3120772 | 3121632 | ComQ | S | A |  |
|  |  |  |  |  |  |  |  |  |
| ***B. subtilis* RO_NN_1** | 384176765 | - | 3043640 | 3044284 | ComA | S | A | [1–5]^4^ |
|  | 384176766 | - | 3044365 | 3046572 | ComP | S | A |  |
| NC_017195 | 384176767 | - | 3046670 | 3046831 | ComX | S | A |  |
|  | 384176768 | - | 3046846 | 3047706 | ComQ | S | A |  |
|  |  |  |  |  |  |  |  |  |
| ***B. subtilis* natto BEST195** | 428280660 | - | 3015448 | 3016092 | ComA | S | A | [4]^4^ |
|  | 428280661 | - | 3016173 | 3018485 | ComP | S | A |  |
| NC_017196 | 428280662 | - | 3018501 | 3018722 | ComX | S | A |  |
|  | 428280663 | - | 3018724 | 3019587 | ComQ | S | A |  |
|  |  |  |  |  |  |  |  |  |
| ***B. subtilis* BSn5** | 321312713 | - | 1260169 | 1260813 | ComA | S |  | [1–5]^4^ |
|  | 321312714 | - | 1260894 | 1263206 | ComP | S |  |  |
| NC_014976 | 321312715 | - | 1263222 | 1263443 | ComX | S | B |  |
|  | 321312716 | - | 1263440 | 1264309 | ComQ | S | B |  |
|  |  |  |  |  |  |  |  |  |
| ***B. subtilis* 168** | 16080219 | - | 3252804 | 3253448 | ComA | S |  |  |
|  | 255767724 | - | 3253529 | 3255838 | ComP | S |  | [2] |
| NC_000964 | 16080221 | - | 3255853 | 3256020 | ComX | S | B |  |
| **­­­­** | 255767725 | - | 3256008 | 3256907 | ComQ | S | B |  |
|  |  |  |  |  |  |  |  |  |
| ***B. subtilis* BSP1** | 430757775 | + | 970082 | 970981 | ComQ | S | B |  |
|  | 430757774 | + | 970969 | 971136 | ComX | S | B | [1–5]^4^ |
| NC_019896 | 430757773 | + | 971151 | 973460 | ComP | S |  |  |
|  | 430757772 | + | 973541 | 974185 | ComA | S |  |  |
|  |  |  |  |  |  |  |  |  |
| ***B. subtilis* QB928** | 402777326 | - | 3184243 | 3184887 | ComA | S |  | [1–5]^4^ |
|  | 402777327 | - | 3184968 | 3187277 | ComP | S |  |  |
| NC_018520 | 402777328 | - | 3187292 | 3187459 | ComX | S | B |  |
|  | 402777329 | - | 3187447 | 3188346 | ComQ | S | B |  |
|  |  |  |  |  |  |  |  |  |
| ***B. subtilis* subsp. subtilis str. BAB-1** | 472331527 | - | 3027997 | 3028641 | ComA | S | A |  |
|  | 472331528 | - | 3028722 | 3031022 | ComP | S | A | [1–5]^4^ |
| NC_020832 | 472331529 | - | 3031034 | 3031198 | ComX | S | A |  |
|  | 472331530 | - | 3031211 | 3032071 | ComQ | S | A |  |
|  |  |  |  |  |  |  |  |  |
| ***B. subtilis* spizizenii W23** | 305675755 | - | 3036239 | 3036883 | ComA | S | A | [3]^5^ |
|  | 305675756 | - | 3036964 | 3039288 | ComP | S | A |  |
| NC_014479 | 305675757 | - | 3039296 | 3039460 | ComX | S | A |  |
|  | 305675758 | - | 3039473 | 3040333 | ComQ | S | A |  |
|  |  |  |  |  |  |  |  |  |
| ***B. subtilis* spizizenii TU B_10** | 350267360 | - | 3194765 | 3195409 | ComA | S |  | [3]^5^ |
|  | 350267361 | - | 3195490 | 3197802 | ComP | S |  |  |
| NC_016047 | 350267362 | - | 3197818 | 3198039 | ComX | S | B |  |
|  | 350267363 | - | 3198036 | 3198905 | ComQ | S | B |  |
|  |  |  |  |  |  |  |  |  |
| ***B. pumilus* SAFR_032** | 157693590 | - | 2835144 | 2835785 | ComA | S |  |  |
|  | 157693591 | - | 2835866 | 2838172 | ComP | S |  |  |
| NC_009848 | 157693592 | - | 2838186 | 2838356 | ComX | S | B |  |
|  | 157693593 | - | 2838334 | 2839257 | ComQ | S | B |  |
|  |  |  |  |  |  |  |  |  |
| ***B. amyloliquefaciens* FZB42** | 154687277 | - | 2994495 | 2995139 | ComA | S |  | [9]^5^ |
|  | 154687278 | - | 2995220 | 2997520 | ComP | S |  |  |
| NC_009725 | 154687279 | - | 2997534 | 2997707 | ComX | S | B |  |
|  | 154687280 | - | 2997676 | 2998536 | ComQ | S | B |  |
|  |  |  |  |  |  |  |  |  |
| ***B. amyloliquefaciens* DSM7** | 308174858 | - | 3026887 | 3027531 | ComA | S | A | [9]^4,5^ |
|  | 308174859 | - | 3027612 | 3029918 | ComP | S | A |  |
| NC_014551 | 308174860 | - | 3029941 | 3030117 | ComX | S | A |  |
|  | 308174861 | - | 3030136 | 3031011 | ComQ | S | A |  |
|  |  |  |  |  |  |  |  |  |
| ***B. amyloliquefaciens* LL3** | 384165616 | - | 3040520 | 3041164 | ComA | S | A | [9]^4,5^ |
|  | 384165617 | - | 3041245 | 3043567^6^ | ComP | S | A |  |
| NC_017190 | 384165618 | - | 3043574 | 3043750 | ComX | S | A |  |
|  | 384165619 | - | 3043769 | 3044644 | ComQ | S | A |  |
|  |  |  |  |  |  |  |  |  |
| ***B. amyloliquefaciens* IT-45** | 451345699 | + | 905489 | 906400 | ComQ | S | A |  |
|  | 451345700 | + | 906400 | 906564 | ComX | S | A | [9]^4,5^ |
| NC_020272 | 451345701 | + | 906576 | 908867 | ComP | S | A |  |
|  | 451345702 | + | 908948 | 909592 | ComA | S | A |  |
|  |  |  |  |  |  |  |  |  |
| ***B. amyloliquefaciens* *plantarum* AS43_3** | 429506438 | - | 3027197 | 3027841 | ComA | S | A | [9]^4,5^ |
|  | 429506439 | - | 3027922 | 3030228 | ComP | S | A |  |
| NC_019842 | 429506440 | - | 3030247 | 3030423 | ComX | S | A |  |
|  | 429506441 | - | 3030550 | 3031314 | ComQ | S | A |  |
|  |  |  |  |  |  |  |  |  |
| ***B. amyloliquefaciens* subsp. *plantarum* UCMB5113** | 530615546 | - | 2987612 | 2988256 | ComA | S |  |  |
|  | 530615547 | - | 2988337 | 2990688 | ComP | S | C | [9]^4,5^ |
| NC_022081 | 530615548 | - | 2990666 | 2990836 | ComX | S | C |  |
|  | 530615549 | - | 2990833 | 2991741 | ComQ | S | C |  |
|  |  |  |  |  |  |  |  |  |
| ***B. amyloliquefaciens subsp. plantarum* UCMB5033** | 530570767 | - | 3144440 | 3145084 | ComA | S | A | [9]^4,5^ |
|  | 530570768 | - | 3145165 | 3147474 | ComP | S | A |  |
| NC_022075 | 530570769 | - | 3147494 | 3147670 | ComX | S | A |  |
|  | 530570770 | - | 3147670 | 3148608 | ComQ | S | A |  |
|  |  |  |  |  |  |  |  |  |
| ***B. amyloliquefaciens* subsp. *plantarum* UCMB5036** | 452856781 | - | 2974358 | 2975002 | ComA | S |  | [9]^4,5^ |
|  | 452856782 | - | 2975083 | 2977434 | ComP | S | C |  |
| NC_020410 | 452856783 | - | 2977412 | 2977582 | ComX | S | C |  |
|  | 452856784 | - | 2977579 | 2978508 | ComQ | S | C |  |
|  |  |  |  |  |  |  |  |  |
| ***B. atrophaeus* 1942** | 311069668 | - | 2685134 | 2685775 | ComA | S |  |  |
|  | 311069669 | - | 2685857 | 2688166 | ComP | S |  |  |
| NC_014639 | 311069670 | - | 2688182 | 2688403 | ComX | S | B |  |
|  | 311069671 | - | 2688400 | 2689269 | ComQ | S | B |  |
|  |  |  |  |  |  |  |  |  |
| ***Syntrophobotulus glycolicus* DSM_8271** | 325289007 | - | 806051 | 806701 | ComA | N | A |  |
|  | 325289008 | - | 806717 | 809059 | ComP | N | A |  |
| NC_015172 | 325289009 | - | 809121 | 809282 | ComX | N | A |  |
|  | 325289010 | - | 809393 | 810307 | ComQ | N | A |  |
|  |  |  |  |  |  |  |  |  |
| ***Desulfosporosinus meridiei* DSM_13257** | 402572493 | + | 2120062 | 2120976 | ComQ | N | A |  |
|  | 402572494 | + | 2121084 | 2121248 | ComX | N | A |  |
| NC_018515 | 402572495 | + | 2121307 | 2123646 | ComP | N | A |  |
|  | 402572496 | + | 2123662 | 2124318 | ComA | N | A |  |
|  |  |  |  |  |  |  |  |  |
| ***Lysinibacillus sphaericus* C3_41** | 169829964 | - | 4400921 | 4401511 | ComA | N | A |  |
|  | 169829965 | - | 4401537 | 4403717 | ComP | N | A |  |
| NC_010382 | 169829966 | - | 4403791 | 4403973 | ComX | N | A |  |
|  | 169829967 | - | 4403984 | 4404847 | ComQ | N | A |  |
|  |  |  |  |  |  |  |  |  |
| ***Lysinibacillus sphaericus* C3_41** | 169829784 | - | 4235148 | 4235750 | ComA | N | D |  |
|  | 169829785 | - | 4235743 | 4237965 | ComP | N | D |  |
| NC_010382 | 169829786 | - | 4238037 | 4238201 | ComX | N |  |  |
|  | 169829787 | - | 4238216 | 4239109 | ComQ | N |  |  |
|  |  |  |  |  |  |  |  |  |
| ***B. licheniformis* 9945A** | 511064159 | - | 3397421 | 3398059 | ComA | S |  | [6]^5^, [7,8]^4^ |
|  | 511064160 | - | 3398146 | 3400461 | ComP | S |  |  |
| NC_021362 | 511064161 | - | 3400482 | 3400652 | ComX | S | B |  |
|  | 511064162 | - | 3400624 | 3401535 | ComQ | S | B |  |
|  |  |  |  |  |  |  |  |  |
| ***Geobacillus* C56 T3** | 297528712 | + | 359832 | 360671 | ComQ | N | A |  |
|  | 297528713 | + | 360691 | 360846 | ComX | N | A |  |
| NC_014206 | 297528714 | + | 360874 | 363117 | ComP | N | A |  |
|  | 297528715 | + | 363279 | 363950 | ComA | N | A |  |
|  |  |  |  |  |  |  |  |  |
| ***Geobacillus thermoglucosidasius* C56_YS93** | 336236914 | - | 3536986 | 3537657 | ComA | N | A |  |
|  | 336236915 | - | 3537683 | 3539947 | ComP | N | A |  |
| NC_015660 | 336236916 | - | 3539954 | 3540109 | ComX | N | A |  |
|  | 336236917 | - | 3540129 | 3541061 | ComQ | N | A |  |
|  |  |  |  |  |  |  |  |  |
| ***Anoxybacillus flavithermus* WK1** | 212638393 | + | 589585 | 590475 | ComQ | N |  |  |
|  | 212638394 | + | 590494 | 590652 | ComX | N |  |  |
| NC_011567 | 212638395 | + | 590656 | 592917 | ComP | N | D |  |
|  | 212638396 | + | 592857 | 593594 | ComA | N | D |  |
|  |  |  |  |  |  |  |  |  |
| ***B. coagulans* 36D1** | 347752472 | + | 2267386 | 2268255 | ComQ | N | A |  |
|  | 347752473 | + | 2268362 | 2268523 |  | N | A |  |
| NC_016023 | 347752474 | + | 2268530 | 2270809 | ComP | N | A |  |
|  | 347752475 | + | 2270837 | 2271490 | ComA | N | A |  |

^1^Clades are indicated as S (*Bacillus Subtilis* type) or N (non-B. subtilis type)

^2^Overlap types (A-E) are indicated in Figure 3

^3^Evidence is indicated with the number of reference, unless a superscript (4 or 5) indicates it otherwise.

^4^experimentally tested on a different strain of the same subspecies/species

^5^ assumption based on sequence similarity

^6^ Corrected value based on HMM run on DNA sequences and verified by six frame translation. The original CDS annotation in the database starts at position 3042795. We assumed that this was a database error since the actual reading frame was intact.

**References:**

1. Magnuson R, Solomon J, Grossman AD (1994) Biochemical and genetic characterization of a competence pheromone from B. subtilis. Cell 77: 207–216.

2. Ansaldi M, Marolt D, Stebe T, Mandic-Mulec I, Dubnau D (2002) Specific activation of the Bacillus quorum-sensing systems by isoprenylated pheromone variants. Mol Microbiol 44: 1561–1573.

3. Stefanic P, Decorosi F, Viti C, Petito J, Cohan FM, et al. (2012) The quorum sensing diversity within and between ecotypes of Bacillus subtilis. Environ Microbiol 14: 1378–1389. doi:10.1111/j.1462-2920.2012.02717.x.

4. Stefanic P, Mandic-Mulec I (2009) Social interactions and distribution of Bacillus subtilis pherotypes at microscale. J Bacteriol 191: 1756–1764. doi:10.1128/JB.01290-08.

5. Tortosa P, Logsdon L, Kraigher B, Itoh Y, Mandic-Mulec I, et al. (2001) Specificity and genetic polymorphism of the Bacillus competence quorum-sensing system. J Bacteriol 183: 451–460. doi:10.1128/JB.183.2.451-460.2001.

6. Lapidus A, Galleron N, Andersen JT, Jørgensen PL, Ehrlich SD, et al. (2002) Co-linear scaffold of the Bacillus licheniformis and Bacillus subtilis genomes and its use to compare their competence genes. FEMS Microbiol Lett 209: 23–30.

7. Daniele De Vizio (2011) Investigation of quorum sensing process in Bacillus licheniformis.

8. Hoffmann K, Wollherr A, Larsen M, Rachinger M, Liesegang H, et al. (2010) Facilitation of direct conditional knockout of essential genes in Bacillus licheniformis DSM13 by comparative genetic analysis and manipulation of genetic competence. Appl Environ Microbiol 76: 5046–5057. doi:10.1128/AEM.00660-10.

9. Chen XH, Koumoutsi A, Scholz R, Eisenreich A, Schneider K, et al. (2007) Comparative analysis of the complete genome sequence of the plant growth-promoting bacterium Bacillus amyloliquefaciens FZB42. Nat Biotechnol 25: 1007–1014. doi:10.1038/nbt1325.
