# Supplementary material for: ComQXPA Quorum Sensing Systems May Not Be Unique to Bacillus subtilis: A Census in Prokaryotic Genomes
Source: PLoS One. 2014 May 2;9(5):e96122. doi: 10.1371/journal.pone.0096122 (PMC4008528; doi:10.1371/journal.pone.0096122)
Supplement: Table S2 — List of protein sequence sets used for building HMM recognizers. (DOCX) [file pone.0096122.s006.docx]

**Table 2: List of protein sequence sets used for building HMM recognizers**

| **ComP Protein ids** | |
| --- | --- |
| Q99027 | Bacillus subtilis (strain 168) GN |
| Q9LC68 | Bacillus subtilis GN |
| C3CRM3 | Bacillus thuringiensis Bt407 GN |
| J7JZ80 | Bacillus subtilis QB928 GN |
| M4KUZ5 | Bacillus subtilis XF-1 GN |
| F4EL12 | Bacillus amyloliquefaciens GN |
| N0DFC8 | Bacillus subtilis BEST7003 GN |
| I0UK07 | Bacillus licheniformis WX-02 GN |
| E1LV14 | Streptococcus mitis SK597 GN |
| M1XDA6 | Bacillus amyloliquefaciens subsp. plantarum UCMB5036 GN |
| D0UX73 | Bacillus subtilis GN |
| E5W7I0 | Bacillus sp. BT1B_CT2 GN |
| D9YRL1 | Bacillus licheniformis 9945A GN |
| H8XGD5 | Bacillus amyloliquefaciens subsp. plantarum YAU B9601-Y2 GN |
| I4VB26 | Bacillus sp. M 2-6 GN |
| Q9K5K7 | Bacillus mojavensis PE |
| L8ATH1 | Synechocystis sp. PCC 6803 GN |
| D4G0R2 | Bacillus subtilis subsp. natto BEST195 GN |
| G4P057 | Bacillus subtilis subsp. spizizenii TU-B-10 GN |
| K2NMF6 | Bacillus sp. HYC-10 GN |
| H2ACI8 | Bacillus amyloliquefaciens subsp. plantarum CAU B946 GN |
| A8FGX6 | Bacillus pumilus (strain SAFR-032) GN |
| B1HMR5 | Lysinibacillus sphaericus (strain C3-41) GN |
| B9IRA2 | Bacillus cereus (strain Q1) GN |
| E1UP81 | Bacillus amyloliquefaciens (strain ATCC 23350 / DSM 7 / BCRC 11601 / NBRC 15535 / NRRL B-14393) GN |
| E0TZW2 | Bacillus subtilis subsp. spizizenii (strain ATCC 23059 / NRRL B-14472 / W23) GN |
| Q63FE5 | Bacillus cereus (strain ZK / E33L) GN |
| C0Z7M3 | Brevibacillus brevis (strain 47 / JCM 6285 / NBRC 100599) GN |

| **ComQ protein ids** | |
| --- | --- |
| P33690 | Bacillus subtilis (strain 168) GN |
| Q38HY5 | Bacillus mojavensis GN |
| C0JBL2 | Bacillus subtilis GN |
| C0JBL3 | Bacillus subtilis GN |
| C0JBM1 | Bacillus subtilis GN |
| Q9R9J5 | Bacillus subtilis GN |
| Q8VLM0 | Bacillus subtilis GN |
| A8D459 | Bacillus subtilis GN |
| C0JBL9 | Bacillus subtilis GN |
| C0JBM9 | Bacillus subtilis GN |
| Q9K5L3 | Bacillus subtilis GN |
| Q8VQ63 | Bacillus licheniformis GN |
| J7JYM2 | Bacillus subtilis QB928 GN |
| A3KLB5 | Bacillus amyloliquefaciens GN |
| F4E8Z8 | Bacillus amyloliquefaciens TA208 GN |
| F4EL14 | Bacillus amyloliquefaciens GN |
| N0DIG3 | Bacillus subtilis BEST7003 GN |
| L8PVG2 | Bacillus subtilis subsp. inaquosorum KCTC 13429 GN |
| A3I6I9 | Bacillus sp. B14905 GN |
| M1UJB8 | Bacillus subtilis subsp. subtilis 6051-HGW GN |
| M5PBH3 | Bacillus sonorensis L12 GN |
| I0UK09 | Bacillus licheniformis WX-02 GN |
| G0IJZ8 | Bacillus amyloliquefaciens XH7 GN |
| M1X7L9 | Bacillus amyloliquefaciens subsp. plantarum UCMB5036 GN |
| Q306U1 | Bacillus subtilis GN |
| Q8VQF0 | Bacillus subtilis GN |
| C0JBL1 | Bacillus subtilis GN |
| C0JBL7 | Bacillus subtilis GN |
| E5W7I2 | Bacillus sp. BT1B_CT2 GN |
| C0JBN8 | Bacillus subtilis GN |
| Q8VQE0 | Bacillus subtilis GN |
| C0JBP4 | Bacillus subtilis GN |
| C0JBM5 | Bacillus subtilis GN |
| C0JBM6 | Bacillus subtilis GN |
| Q8VQE2 | Bacillus subtilis GN |
| C0JBN0 | Bacillus subtilis GN |
| Q8VQE8 | Bacillus subtilis GN |
| C0JBP3 | Bacillus subtilis GN |
| C0JBN7 | Bacillus subtilis GN |
| Q8VQF3 | Bacillus subtilis GN |
| C0JBP1 | Bacillus subtilis GN |
| D9YRK9 | Bacillus licheniformis 9945A GN |
| C0JBP5 | Bacillus subtilis GN |
| C0JBN9 | Bacillus subtilis GN |
| C0JBP6 | Bacillus subtilis GN |
| Q9FAF1 | Bacillus subtilis GN |
| Q38HY3 | Bacillus subtilis GN |
| G9LQ79 | Bacillus subtilis subsp. subtilis GN |
| H8XGD6 | Bacillus amyloliquefaciens subsp. plantarum YAU B9601-Y2 GN |
| Q306U7 | Bacillus mojavensis GN |
| Q2XTA3 | Bacillus mojavensis GN |
| Q8VQE4 | Bacillus mojavensis GN |
| Q38HY0 | Bacillus mojavensis GN |
| Q9K5K9 | Bacillus mojavensis PE |
| Q8VQE6 | Bacillus mojavensis GN |
| D7WXF7 | Lysinibacillus fusiformis ZC1 GN |
| R7ZHL6 | Lysinibacillus sphaericus OT4b.31 GN |
| L8AUK7 | Synechocystis sp. PCC 6803 GN |
| G4P059 | Bacillus subtilis subsp. spizizenii TU-B-10 GN |
| G4ERS5 | Bacillus subtilis subsp. subtilis str. SC-8 GN |
| K9ARW3 | Lysinibacillus fusiformis ZB2 GN |
| M5JIA4 | Anoxybacillus flavithermus TNO-09.006 GN |
| M1KGU1 | Bacillus amyloliquefaciens IT-45 GN |
| I2HUS5 | Bacillus sp. 5B6 GN |
| L0D2T5 | Bacillus subtilis subsp. subtilis str. BSP1 GN |
| H2ACI9 | Bacillus amyloliquefaciens subsp. plantarum CAU B946 GN |
| G4P3U8 | Bacillus subtilis subsp. subtilis str. RO-NN-1 GN |
| D5MYQ3 | Bacillus subtilis subsp. spizizenii ATCC 6633 GN |
| B1HMR7 | Lysinibacillus sphaericus (strain C3-41) GN |
| A7Z884 | Bacillus amyloliquefaciens (strain FZB42) GN |
| A4IQ13 | Geobacillus thermodenitrificans (strain NG80-2) GN |
| B9J4N7 | Bacillus cereus (strain Q1) GN |
| E1UP83 | Bacillus amyloliquefaciens (strain ATCC 23350 / DSM 7 / BCRC 11601 / NBRC 15535 / NRRL B-14393) GN |
| E0TZW4 | Bacillus subtilis subsp. spizizenii (strain ATCC 23059 / NRRL B-14472 / W23) GN |
| B7GGX3 | Anoxybacillus flavithermus (strain DSM 21510 / WK1) GN |

| **ComX Protein ids** | |
| --- | --- |
| P0CY50 | Bacillus subtilis GN |
| P0CY51 | Bacillus subtilis subsp. spizizenii GN |
| P45453 | Bacillus subtilis (strain 168) GN |
| Q9K5K2 | Bacillus mojavensis GN |
| Q9K5K3 | Bacillus mojavensis GN |
| A8D470 | Bacillus subtilis GN |
| Q9R9J4 | Bacillus subtilis GN |
| I2DA18 | Bacillus subtilis GN |
| A8D477 | Bacillus subtilis GN |
| Q9K5L2 | Bacillus subtilis GN |
| Q9FAF0 | Bacillus subtilis GN |
| Q9K5K4 | Bacillus subtilis GN |
| I2D9Y4 | Bacillus subtilis GN |
| Q8VLG1 | Bacillus subtilis GN |
| J7JR85 | Bacillus subtilis QB928 GN |
| F4E8Z7 | Bacillus amyloliquefaciens TA208 GN |
| A3KLB6 | Bacillus amyloliquefaciens GN |
| F4EL13 | Bacillus amyloliquefaciens GN |
| K2IKV1 | Bacillus amyloliquefaciens subsp. plantarum M27 GN |
| N0DJ43 | Bacillus subtilis BEST7003 GN |
| M1TDX0 | Bacillus subtilis subsp. subtilis 6051-HGW GN |
| I0UK08 | Bacillus licheniformis WX-02 GN |
| M1XCL6 | Bacillus amyloliquefaciens subsp. plantarum UCMB5036 GN |
| I2D9X2 | Bacillus subtilis GN |
| E5W7I1 | Bacillus sp. BT1B_CT2 GN |
| G9LQ80 | Bacillus subtilis subsp. subtilis GN |
| I4VB27 | Bacillus sp. M 2-6 GN |
| M2W8Q4 | Bacillus subtilis MB73/2 GN |
| Q2XTA2 | Bacillus mojavensis GN |
| H5Y5H0 | Desulfosporosinus youngiae DSM 17734 GN |
| L8AUK1 | Synechocystis sp. PCC 6803 GN |
| D4G0R3 | Bacillus subtilis subsp. natto BEST195 GN |
| G4P058 | Bacillus subtilis subsp. spizizenii TU-B-10 GN |
| Q8VQ62 | Bacillus licheniformis GN |
| K2P4K5 | Bacillus sp. HYC-10 GN |
| K1L2C5 | Bacillus isronensis B3W22 GN |
| L0D226 | Bacillus subtilis subsp. subtilis str. BSP1 GN |
| Q65FH5 | Bacillus licheniformis (strain DSM 13 / ATCC 14580) GN |
| D4YAC7 | Geobacillus thermoglucosidasius (strain C56-YS93) GN |
| A8FGX7 | Bacillus pumilus (strain SAFR-032) GN |
| J7IZ32 | Desulfosporosinus meridiei (strain ATCC BAA-275 / DSM 13257 / NCIMB 13706 / S10) GN |
| E0TZW3 | Bacillus subtilis subsp. spizizenii (strain ATCC 23059 / NRRL B-14472 / W23) GN |
| E3IGW5 | Geobacillus sp. (strain Y4.1MC1) GN |
| D7CZV6 | Geobacillus sp. (strain C56-T3) GN |

| **ComA protein ids** | |
| --- | --- |
| P14204 | Bacillus subtilis (strain 168) |
| Q00828 | Bacillus subtilis (strain 168) |
| C3CRM4 | Bacillus thuringiensis Bt407 |
| J7JSC1 | Bacillus subtilis QB928 |
| L0BQ19 | Bacillus amyloliquefaciens subsp. plantarum AS43.3 |
| A3KLB8 | Bacillus amyloliquefaciens |
| F4E8Z6 | Bacillus amyloliquefaciens TA208 |
| F4EL11 | Bacillus amyloliquefaciens |
| F2H1N4 | Bacillus thuringiensis serovar chinensis CT-43 |
| C3DSN6 | Bacillus thuringiensis serovar sotto str. T04001 |
| C2N8X0 | Bacillus cereus ATCC 10876 |
| C3EAV5 | Bacillus thuringiensis serovar pakistani str. T13001 |
| C3DZP2 | Bacillus thuringiensis serovar pakistani str. T13001 |
| K2HS56 | Bacillus amyloliquefaciens subsp. plantarum M27 |
| N0DGZ9 | Bacillus subtilis BEST7003 |
| M1U5V1 | Bacillus subtilis subsp. subtilis 6051-HGW |
| C3D9H0 | Bacillus thuringiensis serovar thuringiensis str. T01001 |
| I0U5B5 | Geobacillus thermoglucosidans TNO-09.020 |
| G0IJZ6 | Bacillus amyloliquefaciens XH7 |
| F3M8J0 | Paenibacillus sp. HGF5 |
| C2RFQ1 | Bacillus cereus m1550 |
| C3I8K9 | Bacillus thuringiensis IBL 200 |
| M1XIH9 | Bacillus amyloliquefaciens subsp. plantarum UCMB5036 |
| E5W7H9 | Bacillus sp. BT1B_CT2 |
| Q7DIK8 | Bacillus subtilis |
| D9YRL2 | Bacillus licheniformis 9945A |
| H8XGD4 | Bacillus amyloliquefaciens subsp. plantarum YAU B9601-Y2 |
| M2UEA9 | Bacillus subtilis MB73/2 |
| R8GM28 | Bacillus cereus BAG1X2-3 |
| R8T2Z0 | Bacillus cereus VD140 |
| M4XCR6 | Bacillus subtilis subsp. subtilis str. BAB-1 |
| L8AQZ9 | Synechocystis sp. PCC 6803 |
| C3FSV2 | Bacillus thuringiensis serovar berliner ATCC 10792 |
| D4G0R1 | Bacillus subtilis subsp. natto (strain BEST195) |
| G4P056 | Bacillus subtilis subsp. spizizenii (strain TU-B-10) |
| I2C9K8 | Bacillus amyloliquefaciens Y2 |
| F5LM87 | Paenibacillus sp. HGF7 |
| R8KIF5 | Bacillus cereus BAG2O-1 |
| M5JG39 | Anoxybacillus flavithermus TNO-09.006 |
| D9YRJ9 | Bacillus licheniformis |
| Q8VQ61 | Bacillus licheniformis |
| I0BRU8 | Paenibacillus mucilaginosus K02 |
| R8FJB1 | Bacillus cereus BAG1X2-2 |
| K2N9Q4 | Bacillus sp. HYC-10 |
| R8FXZ0 | Bacillus cereus BAG1X2-1 |
| M1KZG1 | Bacillus amyloliquefaciens IT-45 |
| H6NR67 | Paenibacillus mucilaginosus 3016 |
| K1KQJ7 | Bacillus isronensis B3W22 |
| I2HUS2 | Bacillus sp. 5B6 |
| L0D282 | Bacillus subtilis subsp. subtilis str. BSP1 |
| R8DPB5 | Bacillus cereus BAG1X1-1 |
| R8GY09 | Bacillus cereus VD196 |
| J0LW05 | Bacillus sp. 916 |
| H2ACI7 | Bacillus amyloliquefaciens subsp. plantarum CAU B946 |
| G4P3U5 | Bacillus subtilis subsp. subtilis str. RO-NN-1 |
| Q65FI0 | Bacillus licheniformis (strain DSM 13 / ATCC 14580) |
| A8FGX5 | Bacillus pumilus (strain SAFR-032) |
| F8FNV9 | Paenibacillus mucilaginosus (strain KNP414) |
| Q6HM49 | Bacillus thuringiensis subsp. konkukian (strain 97-27) |
| B1HZD2 | Lysinibacillus sphaericus (strain C3-41) |
| B1HYT9 | Lysinibacillus sphaericus (strain C3-41) |
| A7Z881 | Bacillus amyloliquefaciens (strain FZB42) |
| A4IQ11 | Geobacillus thermodenitrificans (strain NG80-2) |
| B9J4N9 | Bacillus cereus (strain Q1) |
| B9IRA3 | Bacillus cereus (strain Q1) |
| B9IX24 | Bacillus cereus (strain Q1) |
| E1UP80 | Bacillus amyloliquefaciens (strain ATCC 23350 / DSM 7 / BCRC 11601 / NBRC 15535 / NRRL B-14393) |
| E0TZW1 | Bacillus subtilis subsp. spizizenii (strain ATCC 23059 / NRRL B-14472 / W23) |
| B7GGX6 | Anoxybacillus flavithermus (strain DSM 21510 / WK1) |
| Q81HD3 | Bacillus cereus (strain ATCC 14579 / DSM 31) |
| Q63FE4 | Bacillus cereus (strain ZK / E33L) |
| D5TRR5 | Bacillus thuringiensis (strain BMB171) |
| C0Z7M4 | Brevibacillus brevis (strain 47 / JCM 6285 / NBRC 100599) |
