# Supplementary material for: ComQXPA Quorum Sensing Systems May Not Be Unique to Bacillus subtilis: A Census in Prokaryotic Genomes
Source: PLoS One. 2014 May 2;9(5):e96122. doi: 10.1371/journal.pone.0096122 (PMC4008528; doi:10.1371/journal.pone.0096122)

**Domain architecture of ComP proteins**

**1.) 10 Transmembrane helices = 10 (present in 48 proteins):** *B.amyloliquefaciens* FZB4,*B. pumilus* SAFR 032, *B.subtilis subsp. subtilis* str. 168, *Geobacillus* sp. C56 T, *B.subtilis subsp. spizizenii* str.W2,*B.amyloliquefaciens* DSM7,*B. atrophaeus* 194,*G.thermoglucosidasius* C56 YS9,*B.coagulans* 36D1, *B. subtilis subsp. spizizenii* TU B 10, *Bacillus* sp. JS, Desulfosporosinus meridiei DSM 1325,*B.subtilis* QB928,*B.subtilis subsp.* natto BEST195,*B. amyloliquefaciens* subsp. plantarum AS43.3, *B. subtilis subsp.subtilis* str. BSP1,*B. amyloliquefaciens* IT 45,*B.amyloliquefaciens subsp. plantarum* UCMB5036,*B. subtilis subsp. subtilis* str.BAB1,*B. licheniformis* 9945A,*B. amyloliquefaciens subsp. plantarum* UCMB5033, *B. amyloliquefaciens subsp. plantarum* UCMB5113,*Bacillus sp*. 5B6,*B.sp.* BT1B CT2, *B. subtilis subsp. subtilis* str. SMY, *B.subtilis subsp.subtilis str*. NCIB 3610,*B.subtilis* MB73/2,*B.subtilis subsp. subtilis str.*JH642, *B. subtilis subsp. spizizenii* ATCC 663,*B.cereus* VD10,*B.cereus* BAG4X12 1, *B.cereus* MSX_A, *B.isronensis* B3W2, *B.sonorensis* L12,*B.pumilus* ATCC 706,*B.licheniformis* WX 0, *B.amyloliquefaciens subsp. plantarum* M2, *B.atrophaeus* C89,*B.azotoformans* LMG 9581,*B.mojavensis* RO H 1,*B.vallismortis DV1* F 3,*Geobacillus sp.*G11MC16,*A.flavithermus TNO* 09.006,*L.fusiformis* ZC1,Paenibacillus curdlanolyticus YK9, Desulfosporosinus youngiae DSM 17734)


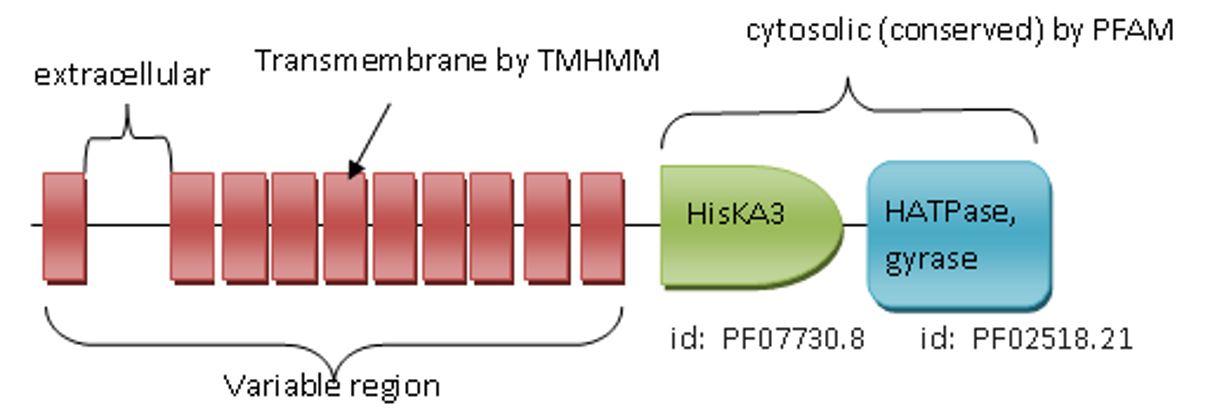


**2.) 9 Transmembrane helices = (present in 11 proteins, red=non-*B.subtilis* clade) :** *L.sphaericus* C341,*A.flavithermus* WK1, *S.glycolicus* DSM 8271,*B.subtilis* BSn5,*B.subtilis subsp. subtilis str*. RONN1,*Bacillus sp.* HYC_10,*Bacillus sp.* M 26,*Bacillus sp.*B14905,*B.subtilis subsp. subtilis str*.SC8, *B.subtilis subsp. inaquosorum* KCTC13429, Anoxybacillus flavithermus TNO 09.006)


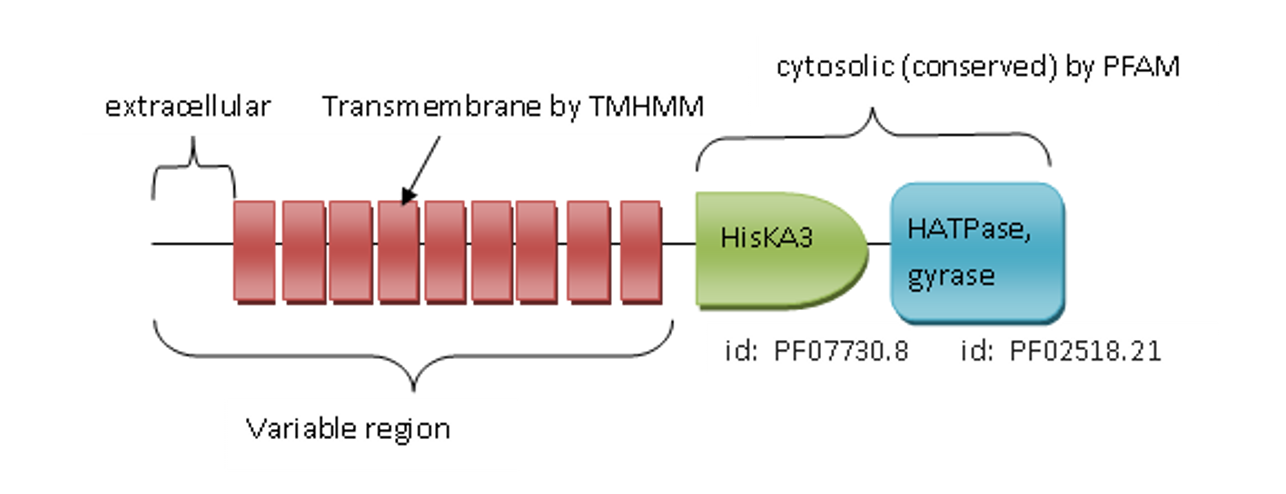

Supplement: Table S3 — Transmembrane domain architecture in ComP proteins. (DOC) [file pone.0096122.s007.doc]
